# Ribosomal RNA Transcription Machineries in Intestinal Protozoan Parasites: A Bioinformatic Analysis

**DOI:** 10.1007/s11686-022-00612-7

**Published:** 2022-08-27

**Authors:** Francisco Alejandro Lagunas-Rangel

**Affiliations:** Department of Surgical Sciences, Uppsala University, Husargatan 3, BMC Box 593, 75124 Uppsala, Sweden

**Keywords:** RNA polymerase I, SL1, UBF, RRN3, POLR1F

## Abstract

**Purpose:**

Ribosome biogenesis is a key process in all living organisms, energetically expensive and tightly regulated. Currently, little is known about the components of the ribosomal RNA (rRNA) transcription machinery that are present in intestinal parasites, such as *Giardia duodenalis*, *Cryptosporidium parvum*, and *Entamoeba histolytica.* Thus, in the present work, an analysis was carried out looking for the components of the rRNA transcription machinery that are conserved in intestinal parasites and if these could be used to design new treatment strategies.

**Methods:**

The different components of the rRNA transcription machinery were searched in the studied parasites with the NCBI BLAST tool in the EuPathDB Bioinformatics Resource Center database. The sequences of the RRN3 and POLR1F orthologs were aligned and important regions identified. Subsequently, three-dimensional models were built with different bioinformatic tools and a structural analysis was performed.

**Results:**

Among the protozoa examined, *C. parvum* is the parasite with the fewest identifiable components of the rRNA transcription machinery. TBP, RRN3, POLR1A, POLR1B, POLR1C, POLR1D, POLR1F, POLR1H, POLR2E, POLR2F and POLR2H subunits were identified in all species studied. Furthermore, the interaction regions between RRN3 and POLR1F were found to be conserved and could be used to design drugs that inhibit rRNA transcription in the parasites studied.

**Conclusion:**

The inhibition of the rRNA transcription machinery in parasites might be a new therapeutic strategy against these microorganisms.

**Supplementary Information:**

The online version contains supplementary material available at 10.1007/s11686-022-00612-7.

## Introduction

Intestinal protozoal infections are a major health problem, especially in developing countries, where poor household hygiene practices, inadequate sanitary facilities, and low socioeconomic conditions favor their spread [1]. In particular, protozoa are responsible for important intestinal diseases in humans, with high morbidity and, in some cases, mortality [2]. *Giardia duodenalis* and *Cryptosporidium parvum* are the most common pathogenic intestinal protozoan parasites with an annual incidence of about 10,000 cases each in the United States and Europe alone, whereas for *Entamoeba histolytica*, a worldwide annual incidence of 100 million cases is estimated [3]. In general, these parasites cause poor digestion, impair absorption and increase nutrient loss, among other things. Indeed, even in asymptomatic infections, subtle damage and disturbances of intestinal function may occur [4]. One aspect to highlight is the increase in treatment failure and the appearance of strains resistant to current drugs due to their massive and inappropriate use, which has led us to the need to devise new treatment strategies [2, 5, 6].

On the other hand, the efficient growth and proliferation of parasites require a balanced production of ribosomes for protein synthesis [7–10]. Notably, the rate-limiting step of ribosome biogenesis is the synthesis of ribosomal RNA (rRNA) by RNA polymerase I (Pol I) [10–12]. The rRNA transcription machinery comprises three main components: the Pol I enzyme, the TBP (TATA-binding protein)-TAF (TBP-associated factor) complex SL1 (selectivity factor 1) and the trans-activator protein UBF (upstream binding factor) [13]. Currently, little is known about the components of the rRNA transcription machinery that are present in intestinal parasites, but it is known that if any of these do not function properly, the parasites die due to cell cycle arrest and apoptosis [14–17]. In this sense, the rRNA transcription machinery becomes a feasible target for the design of new anti-parasitic drugs. The proposal of this work was to identify the putative components of the ribosomal RNA transcription machinery in the three most prominent intestinal protozoan pathogens, *G. duodenalis*, *C. parvum*, and *E. histolytica*. Furthermore, special emphasis was placed on the interaction between RRN3 and POLR1F, which is a key step to link Pol I with the rest of the components of the transcriptional machinery and where anti-parasitic drugs might be designed.

## Materials and Methods

### Database Screening

The amino acid sequences of the proteins involved in the initiation of rRNA transcription in humans were obtained from the UniProt Knowledgebase (UniProtKB) [18] using the name of each protein. To find orthologs of human proteins, the whole genome sequences of the intestinal parasites *G. duodenalis* (Assemblage A_isolate_WB), *C. parvum* (Iowa II) and *E. histolytica* (HM1-IMSS) were examined in the corresponding databases of the EuPathDB Bioinformatics Resource Center [19] and using the NCBI BLAST tool [20] with default search parameters. The rRNA transcription machinery of *Saccharomyces cerevisiae* and the genome of *Entamoeba dispar* were also analyzed for comparative purposes.

### Sequence Analysis

The structural domains of rRNA transcription machinery proteins present in all organisms were predicted and analyzed using InterPro [21]. Multiple sequence alignments of RRN3 orthologs and POLR1F orthologs were performed using Clustal Omega in CLC Genomics Workbench 21 (Qiagen Bioinformatics, Aarhus C, Denmark). Based on these alignments, the identity and similarity percentages between the orthologs were calculated. Three-dimensional structures were predicted using SWISS-MODEL [22], and illustrations were made using UCSF Chimera software [23].

## Results

### TBP is the Only Subunit of the TBP-TAF Complex SL1 that has Identifiable Orthologs in All Species Analyzed. Pol I-Specific Factor RRN3 is also Conserved

The only component of the TBP-TAF complex SL1 that was found in all the organisms analyzed was TBP, with one ortholog in *G. duodenalis*, two in *C. parvum* and three in both *E. histolytica* and *E. parvum*. Since TAF_II_12 orthologs were identified only in *E. histolytica* and *E. dispar*, *G. duodenalis* and *C. parvum* were the species with the fewest identifiable subunits of the SL1 transcription factor. RRN3 orthologs were found in the genome of all organisms, but the sequences of *E. histolytica* and *E. dispar* orthologs diverged widely. Regarding the UBF transcription activator, two orthologs were identified in *G. duodenalis* and three in both *E. histolytica* and *E. dispar*. No orthologs for this protein were found in *C. parvum*. The summary of the results is presented in Tables 1 and 2.

**Table 1:** Prediction of rRNA transcription machineries in *Giardia duodenalis* and *Cryptosporidium parvum*

*Homo sapiens*			*Saccharomyces cerevisiae*			*Gardia duodenalis* (Assemblage A_isolate_WB)					*Cryptosporidium parvum* (Iowa II)				
Gene	UniProtKB^a^	Size^b^	Gene	UniProtKB^a^	Size^b^	Gene	UniProtKB^a^	Size^b^	E-value	*I*^c^	Gene	UniProtKB^a^	Size^b^	E-value	*I*^c^
TBP-TAF complex SL1															
TBP	P62380	339	TBP	P13393	240	GL50803_1721	E2RU70	200	6e−05	25	cgd8_2030 cgd8_210	Q6SEL4 Q5CPZ4	249 195	4e−67 7e−14	51 32
TAF1C	Q15572	869	Rrn6	P32786	894	–	–	–	–	–	–	–	–	–	–
TAF1B	Q53T94	588	Rrn7	P40992	514	–	–	–	–	–	–	–	–	–	–
TAF1A	Q15573	450	Rrn11	Q04712	507	–	–	–	–	–	–	–	–	–	–
TAF1D	Q9H5J8	278	–	–	–	–	–	–	–	–	–	–	–	–	–
TAF12	Q16514	161	–	–	–	–	–	–	–	–	–	–	–	–	–
RRN3	Q9NYV6	651	Rrn3	P36070	627	GL50803_11742	A8B8V3	556	2e−05	23	cgd6_2810	Q5CX25	882	1e−04	27
Transcription activator UBF															
UBF	P17480	764	UAF30	Q08747	228	GL50803_17626 GL50803_3349	A8BW36 A8BDD1	204 183	1e−05 5e−05	30 30	–	–	–	–	–
			Rrn5	Q02983	363						–	–	–	–	–
			Rrn9	P53437	365						–	–	–	–	–
			Rrn10	P38204	145						–	–	–	–	–
RNA polymerase I complex															
POLR1A	O95602	1720	A190	P10964	1664	GL50803_16223 GL50803_23496 GL50803_89347	A8B4F1 A8B7R7 E2RTQ5	1741 2142 2076	1e−78 3e−70 7e−36	28 28 26	cgd3_2620 cgd6_3290 cgd5_730	Q5CUJ2 Q5CWY0 A3FQH3	1895 1871 1805	9e−109 2e−102 1e−70	35 35 33
POLR1B	Q9H9Y6	1135	A135	P22138	1203	GL50803_29436 GL50803_17187 GL50803_17448	A8B827 A8B2N4 E2RU19	1235 1238 1293	3e−129 3e−102 4e−83	34 30 28	cgd1_2770 cgd7_3720 cgd8_170	Q5CSG7 Q5CY45 Q5CPZ8	1281 1177 1287	3e−122 3e−122 5e−121	36 29 29
POLR1C	O15160	346	AC40	P07703	335	GL50803_10055 GL50803_7474	A8B9C8 E2RTY7	350 326	5e−47 5e−16	33 28	cgd8_300 cgd1_2710	Q5CPY6 Q5CSH3	344 355	5e−69 5e−29	40 29
POLR1D	P0DPB6	133	AC19	P28000	142	GL50803_10840	A8B7A8	101	1e−15	59	cgd4_3200	A3FQL8	98	4e−15	60
POLR1E	Q9GZS1	414	A49	Q01080	415	–	–	–	–	–	–	–	–	–	–
POLR1F	Q3B726	338	A43	P46669	326	GL50803_17422	A8BD91	229	1e−03	24	cgd1_1620	Q5CSR5	256	7e−07	27
POLR1G	O15446	510	A34.5	P47006	233	–	–	–	–	–	–	–	–	–	–
POLR1H	Q9P1U0	126	A12.2	P32529	125	GL50803_8518	A8BBB2	103	2e−12	41	cgd7_503 cgd3_2550	F0X620	106 246	7e−08 3e−05	27 41
POLR2E	P19388	210	Rpb5	P20434	215	GL50803_137609 GL50803_8157	E2RU58 E2RU93	229 201	7e−26 5e−16	32 26	cgd2_980	Q5CTZ0	205	7e−62	45
POLR2F	P61218	127	Rpb6	P20435	155	GL50803_15955	E2RTN0	104	4e−26	52	cgd7_4770	Q5CXV6	129	4e−36	72
POLR2H	P52434	150	Rpb8	P20436	146	GL50803_15144	E2RU32	150	1e−07	27	cgd1_2260	Q5CSK9	144	3e−22	35
POLR2K	P53803	58	Rpb12	P40422	70	GL50803_9509	E2RU91	54	1e−04	33	cgd7_3240	A3FPP8	71	9e−12	43
POLR2L	P62875	67	Rpb10	P22139	70	GL50803_14413	A8BAL6	122	1e−22	59	cgd4_3260	A3FQL9	72	1e−28	66
RPA3	P35244	121	RPA14	P50106	137	–	–	–	–	–	–	–	–	–	–

^a^Access number in the UniProt Knowledgebase

^b^Number of amino acids

^c^*I* (Identity) values expressed in percentage (%)

**Table 2:** Prediction of rRNA transcription machineries in *Entamoeba histolytica* and *Entamoeba dispar*

*Homo sapiens*			*Saccharomyces cerevisiae*			*Entamoeba histolytica* (HM1-IMSS)					*Entamoeba dispar* (SAW760)				
Gene	UniProtKB^a^	Size^b^	Gene	UniProtKB^a^	Size^b^	Gene	UniProtKB^a^	Size^b^	E-value	*I*^c^	Gene	UniProtKB^a^	Size^b^	E-value	*I*^c^
TBP-TAF complex SL1															
TBP	P62380	339	TBP	P13393	240	EHI_077240 EHI_112050 EHI_020610	A7UFC2 C4M7H7 P52653	216 212 234	2e−66 2e−64 7e−64	56 56 56	EDI_292240 EDI_260400 EDI_172550	B0EHD5 B0EIX2 B0EIP7	216 212 234	6e−66 1e−64 4e−64	56 56 56
TAF1C	Q15572	869	Rrn6	P32786	894	–	–	–	–	–	–	–	–	–	–
TAF1B	Q53T94	588	Rrn7	P40992	514	–	–	–	–	–	–	–	–	–	–
TAF1A	Q15573	450	Rrn11	Q04712	507	–	–	–	–	–	–	–	–	–	–
TAF1D	Q9H5J8	278	–	–	–	–	–	–	–	–	–	–	–	–	–
TAF12	Q16514	161	–	–	–	EHI_118200 EHI_009490 EHI_118230	C4LZ03 C4LZ05	152 139	5e−07 2e−05	24 26	EDI_264420 EDI_322430 EDI_025860	B0ELF7 B0E5S5	152 139	4e−07 2e−05	24 26
RRN3	Q9NYV6	651	Rrn3	P36070	627	EHI_035130	C4M4Z3	466	3e−25^d^	15	EDI_198050	B0EPH1	466	3e−25^d^	15
Transcription activator UBF															
UBF	P17480	764	UAF30	Q08747	228	EHI_045480 EHI_093800 EHI_179340	C4LTF9 C4LYH1 C4M9X4	111 114 384	3e−06 5e−06 4e−04	31 30 35	EDI_340970 EDI_049480 EDI_110640 EDI_085740	B0EV32 B0EFF8 B0EK44 B0EF23	111 112 395 106	3e−06 3e−06 3e−04 6e−04	33 31 35 40
			Rrn5	Q02983	363										
			Rrn9	P53437	365										
			Rrn10	P38204	145										
RNA polymerase I complex															
POLR1A	O95602	1720	A190	P10964	1664	EHI_095890 EHI_121760 EHI_125350	C4M3E6 Q6IUR3 C4M626	1570 1636 1379	5e−106 5e−106 3e−97	36 30 35	EDI_337480 EDI_169920 EDI_116030	B0EDI6 B0E6R8 B0EG66	1568 1587 1379	4e−102 4e−102 5e−98	36 30 35
POLR1B	Q9H9Y6	1135	A135	P22138	1203	EHI_186020 EHI_095860 EHI_022940	C4MAB3 C4M3E3 C4LUK7	1106 1122 1170	1e−132 1e−132 1e−119	39 31 28	EDI_303700 EDI_337350 EDI_248550	B0ERH0 B0EDI3 B0EG45	1106 1075 1170	2e−124 2e−124 1e−119	40 32 28
POLR1C	O15160	346	AC40	P07703	335	EHI_178010	C4M554	283	5e−24	27	EDI_002920 EDI_044160	B0EJB9 B0EHR6	291 283	1e−73 4e−24	44 27
POLR1D	P0DPB6	133	AC19	P28000	142	EHI_087360	C4LXS9	112	9e−12	34	EDI_085890 EDI_290250	B0E866	112	3e−11	33
POLR1E	Q9GZS1	414	A49	Q01080	415	–	–	–	–	–	–	–	–	–	–
POLR1F	Q3B726	338	A43	P46669	326	EHI_124360	B1N352	212	2e−05	29	EDI_023800	B0EHA3	212	2e−05	28
POLR1G	O15446	510	A34.5	P47006	233	–	–	–	–	–	–	–	–	–	–
POLR1H	Q9P1U0	126	A12.2	P32529	125	EHI_044620 EHI_137900	C4LT84	122	1e−16	40	EDI_323220 EDI_246270	B0EEN5	122	1e−16	40
POLR2E	P19388	210	Rpb5	P20434	215	EHI_142090	C4LW54	204	1e−47	39	EDI_338140	B0EA74	204	6e−48	39
POLR2F	P61218	127	Rpb6	P20435	155	EHI_088230	C4M6S1	122	2e−32	73	EDI_088050 EDI_320930	B0EM77	122	6e−32	72
POLR2H	P52434	150	Rpb8	P20436	146	EHI_038570	C4LZP1	143	6e−15	31	EDI_259550	B0EB74	143	3e−15	31
POLR2K	P53803	58	Rpb12	P40422	70	–	–	–	–	–	–	–	–	–	–
POLR2L	P62875	67	Rpb10	P22139	70	EHI_122780	C4M5M4	73	2e−19	56	–	–	–	–	–
RPA3	P35244	121	RPA14	P50106	137	–	–	–	–	–	–	–	–	–	–

^a^Access number in the UniProt Knowledgebase

^b^Number of amino acids

^c^*I* (Identity) values expressed in percentage (%)

^d^Srivastava *et al*. [31]

### Intestinal Protozoan Parasites Lack Orthologs of the POLR1E, POLR1G, and RPA3 Subunits in RNA Polymerase I Complex

Ortholog search for the 14 major subunits of RNA polymerase I was also performed. Thus, it was found that for the subunits POLR1A, POLR1B, POLR1C, POLR1D, POLR1F, POLR1H, POLR2E, POLR2F and POLR2H there is at least one ortholog in each species. In contrast, for the POLR1E, POLR1G and RPA3 subunits, no orthologs were found in any of the analyzed parasites. Orthologs of the POLR2K subunit were identified in *G. duodenalis* and *C. parvum*, but not in *E. histolytica* and *E. dispar*. Interestingly, no orthologs were found for the POLR2L subunit in *E. dispar*. In this way, *E. dispar* is the organism with the fewest identifiable components of RNA polymerase I. The summary of the results is presented in Tables 1 and 2.

### The Conserved Subunits of the rRNA Transcription Machinery Show Differences Between Them

**Fig. 1:**
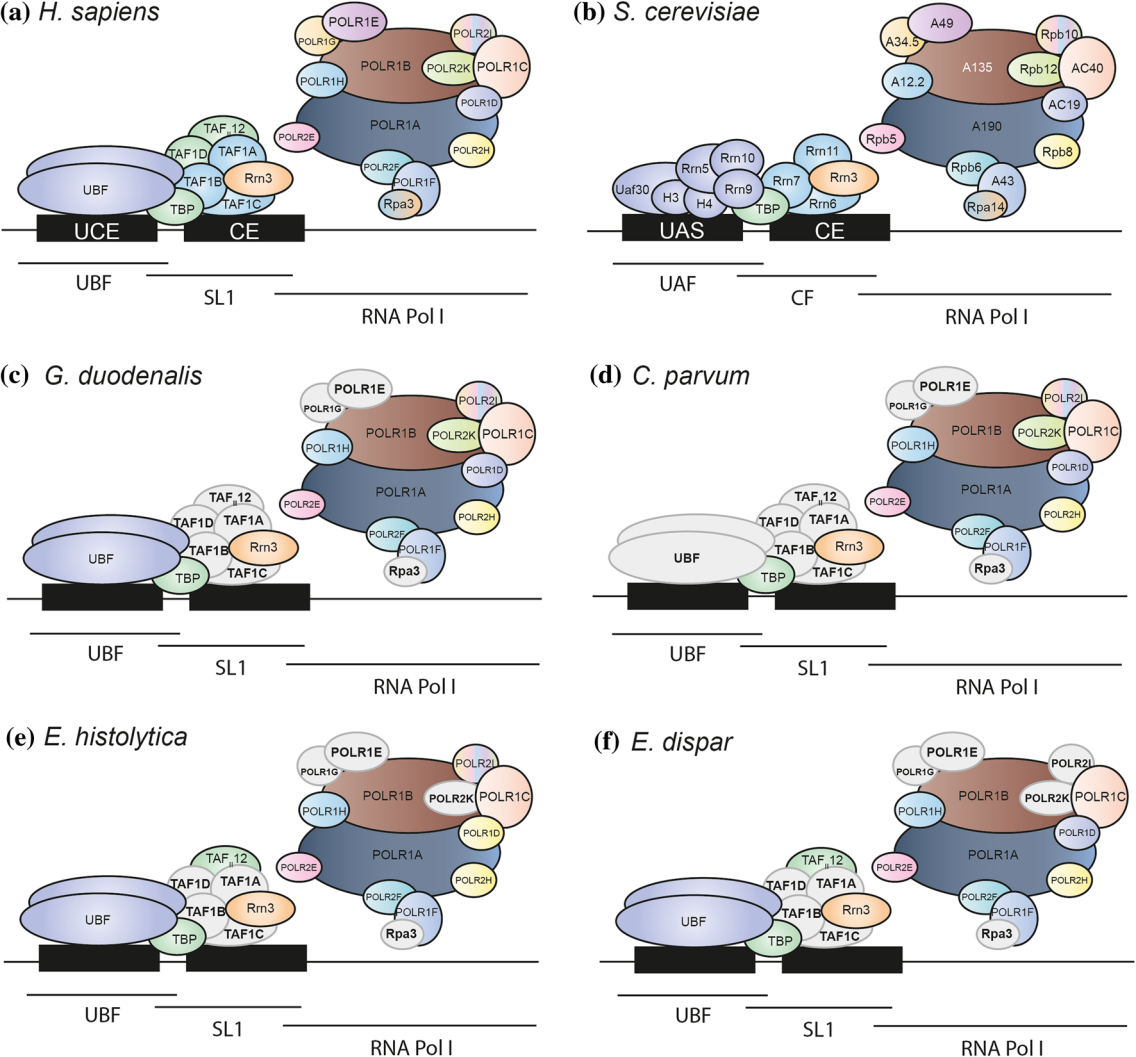
Schematic representation of the rRNA transcription machinery in *Homo sapiens* (**a**), *Saccharomyces cerevisiae* (**b**), *Giardia duodenalis* (**c**), *Cryptosporidium parvum* (**d**), *Entamoeba histolytica* (**e**), and *Entamoeba dispar* (**f**). The rRNA transcription machinery comprises three main components: the Pol I enzyme, the TBP-TAF SL1 complex, and the UBF protein. The TBP-TAF SL1 complex is composed of TBP and at least three TAF subunits, TAF1A, TAF1B and TAF1C (Rrn11, Rrn6 and Rrn7 in yeast). Meanwhile, the human protein UBF replaces the complex formed by H3, H4, Rrn5, Rrn9, Rrn10 and Uaf30 in yeast. Yeast Pol I consists of A190 and A135 (POLR1A and POLR1B in humans) plus Rpb5, Rpb6, Rpb8, Rpb10 and Rpb12 (POLR2E, POLR2F, POLR2H, POLR2L, POLR2K in humans) and the heterodimer AC40-AC19 (POLR1D-POLR1C in humans). The Pol I core is completed with A12.2 (POLR1H in humans), the A43-A14 heterodimer (POLR1F-RPA3 heterodimer in humans) and the A49 and A34.5 subunits (POLR1E and POLR1G in humans). For the subunits appearing in gray, no orthologs were found in the parasites studied

Figure 1 shows the rRNA transcription machinery in each species analyzed according to our bioinformatic analysis. All the orthologs of TBP, POLR1D and POLR1F identified in parasites were proteins smaller than those in humans. The reduction in the number of amino acids was between 26.5% and 42.5% for TBP, between 15.8% and 26.3% for POLR1D, and between 24.3% and 37.3% for POLR1F. Except for *C. parvum*, the RRN3 orthologs had between 14.6% and 28.4% fewer amino acids than the human protein, but RRN3 HEAT repeats are conserved in all species studied. POLR1A orthologs in *G. duodenalis* and *C. parvum* had between 4.9% and 24.5% more amino acids than the human protein, but for the *E. histolytica* and *E. dispar* orthologs the number of amino acids was between 4.9% and 19.8% less than the human counterpart. Meanwhile, POLR1B orthologs from *G. duodenalis* and *C. parvum* were proteins with 6% to 13.4% more amino acids than human protein, but almost all orthologs from *E. histolytica* and *E. dispar* had similar amounts. In contrast, POLR1C orthologs in *G. duodenalis* and *C. parvum* maintained a similar number of amino acids as human protein, but orthologs in *E. histolytica* and *E. dispar* were proteins with 15.9% to 18% less amino acids. Most of the identified orthologs of POLR1H, POLR2E, POLR2F, and POLR2H in parasites were very similar in size to human and yeast proteins. A schematic representation of these data appears in Supplementary Fig. 1.

### The RRN3 and POLR1F Orthologs Maintain Important Residues for Their Interaction in All the Species Studied

**Fig. 2:**
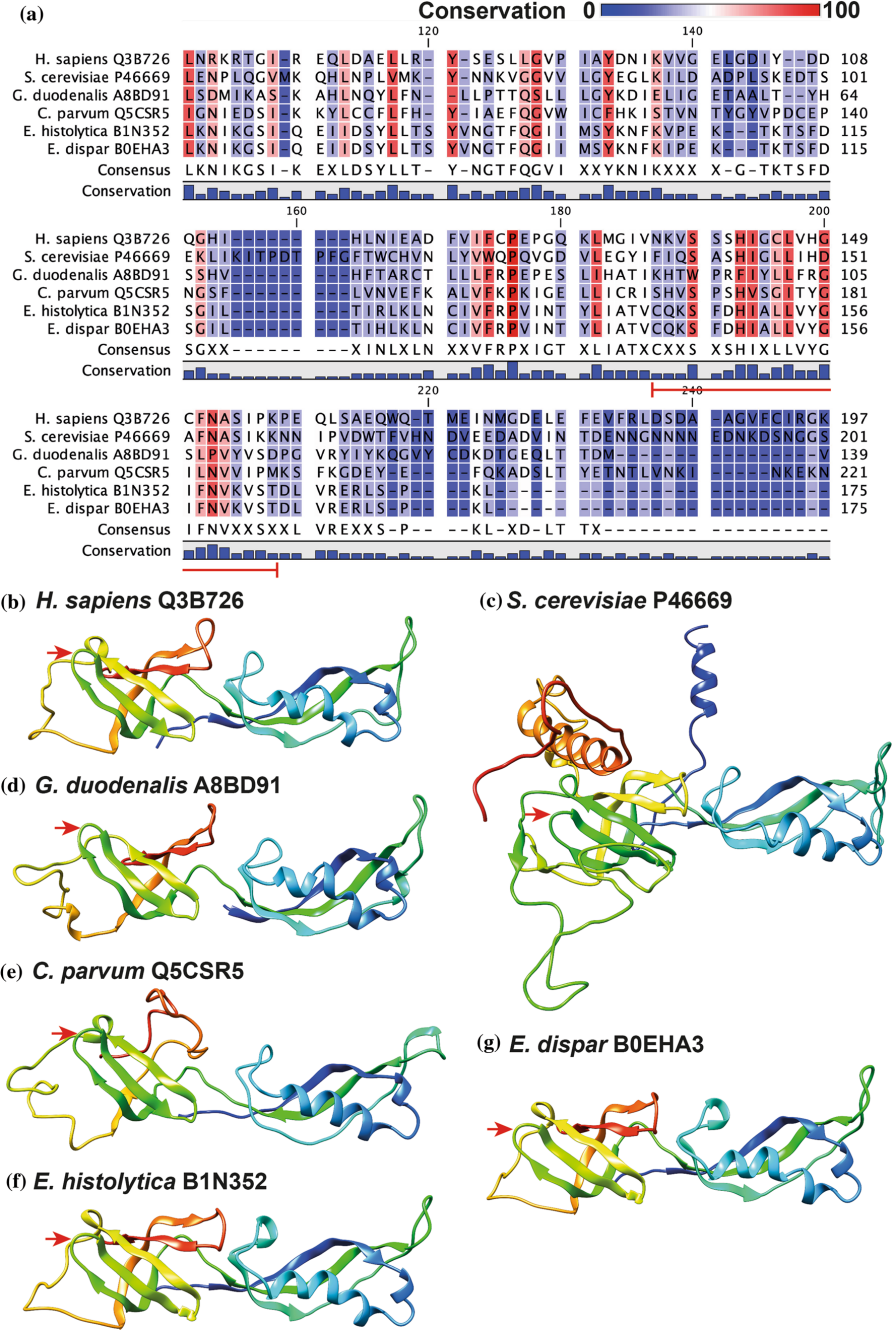
POLR1F orthologs in intestinal parasites preserve the region that interacts with the RRN3 subunit. **A** Sequence alignments of the POLR1F orthologs identified in the different species studied. The degree of conservation is shown in colors and a red line is placed to indicate the amino acids that are known to mediate the interaction of this subunit with RRN3. Predicted structures of human POLR1F (**B**) and their orthologs in *Saccharomyces cerevisiae* (**C**), *Giardia duodenalis* (**D**), *Cryptosporidium parvum* (**E**), *Entamoeba histolytica* (**F**), and *Entamoeba dispar* (**G**). The region that interacts with RRN3 is marked with red arrows (color figure online)

**Fig. 3:**
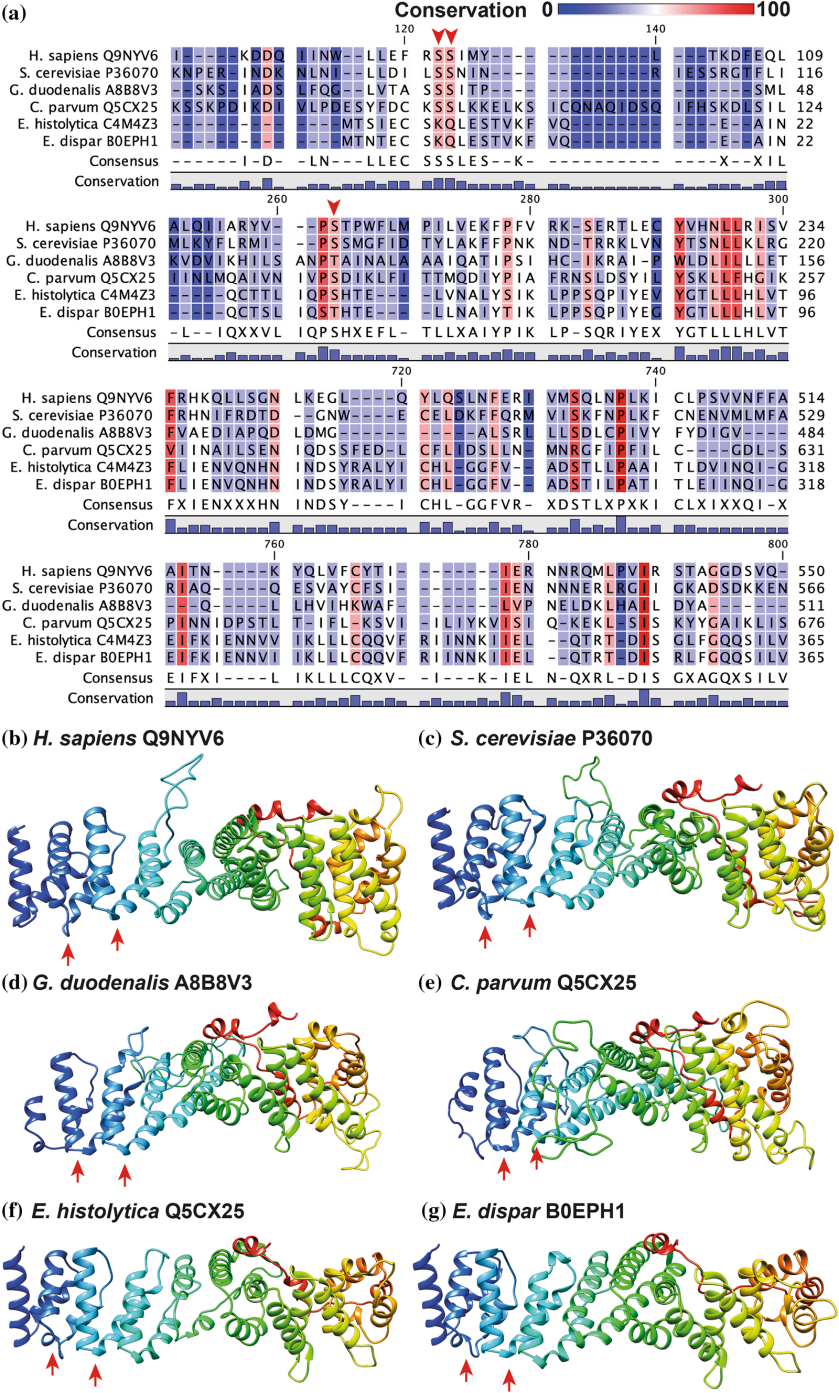
RRN3 orthologs conserve some residues that constitute a serine patch and are known to interact with POLR1F. **A** Sequence alignments of the identified RRN3 orthologs. The degree of conservation is shown in color and arrowheads are placed to indicate the residues that could interact with POLR1F. Predicted structures of human RRN3 (**B**) and their orthologs in *Saccharomyces cerevisiae* (**C**), *Giardia duodenalis* (**D**), *Cryptosporidium parvum* (**E**), *Entamoeba histolytica* (**F**), and *Entamoeba dispar* (**G**). The place where the serine residues that interact with POLR1F are located are marked with red arrows (color figure online)

Despite poor sequence conservation (Table 3), sequence alignments of POLR1F orthologs showed that the region mediating its interaction with the RRN3 subunit is conserved in intestinal protozoan parasites (Fig. 2A). Furthermore, three-dimensional predictions of these parasitic proteins revealed strong structural similarity to their human and yeast counterparts, where the binding area remains exposed in all cases to facilitate their interaction with RRN3 (Fig. 2B). On the other hand, sequence alignment analysis of the RRN3 orthologs also revealed low conservation (Table 3), but residues of a serine patch that serve this protein to bind to POLR1F had high conservation (Fig. 3A), particularly those corresponding to residues S101, S102 and S185 of yeast RRN3. With the three-dimensional predictions, a high structural similarity of the RRN3 orthologs was observed, where the characteristic HEAT repeat fold (repeats of alpha helices joined by a short loop) is maintained. In addition, the identified serine patch residues are exposed in all cases and would allow their interaction with POLR1F orthologs (Fig. 3B). These findings suggest that the interaction points between RRN3 and POLR1F are conserved in different species, including intestinal protozoan parasites.

**Table 3:** Identity and similarity between the orthologs of RRN3 and POLRF1 in the species analyzed

RRN3	*H. sapiens *Q9NYV6	*S. cerevisiae *P36070	*G. duodenalis *A8B8V3	*C. parvum *Q5CX25	*E. histolytica *C4M4Z3	*E. dispar *B0EPH1
*H. sapiens *Q9NYV6		20.06	12.82	11.37	6.09	5.72
*S. cerevisiae *P36070	35.62		13.29	10.11	4.06	4.06
*G. duodenalis *A8B8V3	27.56	27.89		7.89	5.22	4.81
*C. parvum *Q5CX25	23.88	23.05	18.29		8.92	8.92
*E. histolytica *C4M4Z3	12.94	11.53	14.97	17.94		91.20
*E. dispar *B0EPH1	12.56	11.15	14.01	18.05	96.14	

POLR1F	*H. sapiens *Q3B726	*S. cerevisiae *P46669	*G. duodenalis *A8BD91	*C. parvum *Q5CSR5	*E. histolytica *B1N352	*E. dispar *B0EHA3
*H. sapiens *Q3B726		13.98	10.12	13.30	10.54	10.26
*S. cerevisiae *P46669	29.29		10.51	10.40	7.76	8.05
*G. duodenalis *A8BD91	26.01	24.32		7.86	11.15	11.15
*C. parvum *Q5CSR5	23.14	21.07	19.18		13.48	13.48
*E. histolytica *B1N352	20.51	18.97	21.25	27.72		98.58
*E. dispar *B0EHA3	20.51	18.97	21.25	27.72	100	

Cells above and to the right of the central diagonal indicate percent amino acid identity, while cells below and to the left indicate percent similarity

## Discussion

Transcription of rRNA by Pol I is the key regulatory step in ribosome production and is tightly controlled by an intricate network of signaling pathways and epigenetic mechanisms [24]. The transcription by Pol I requires the formation of a preinitiation complex (PIC) that directs promoter-specific transcription of rDNA and whose components are the Pol I enzyme, the TBP-TAF complex SL1 and UBF [25]. The only subunit of the TBP-TAF complex SL1 that was identified in all the species analyzed was TBP. This responds to the fact that TBP is considered the most conserved initiation factor in archaeo-eukaryotic transcription initiation complexes [26]. No orthologs of the three Pol I-specific TAFs were identified, but all species have other members of the TFIID (transcription factor II D) family protein encoded in their genome that could carry out this function. UBF plays an essential role in maintaining a state of euchromatin on rDNA and enhancing rRNA expression [27], and that is why it is interesting that in *C. parvum*, a UBF ortholog was not found. UBF is not essential for the initiation of transcription *in vitro*, but it is essential for the formation of PIC *in vivo* and functions in the pre- and post-initiation steps [25, 27]. However, the genome of *C. parvum* exhibits other proteins with HMG (high mobility group) boxes whose specific function has not yet been characterized. Almost all the core subunits of Pol I were identified in intestinal protozoan parasites, only in *E. histolytica* no orthologs of POLR2K were found and in *E. dispar* orthologs of POLR2K and POLR2L. These last two mentioned subunits are shared with the other two DNA-directed RNA polymerases (RNA Pol I and RNA Pol III). Notably, the heterodimer POLR1E-POLR1G, which is required for RNA elongation by Pol I [28], was not found in any of the analyzed parasite species. Therefore, the question arises how these organisms carry out rRNA elongation. The heterodimer formed by the POLR1F and RPA3 subunits plays a role in recruiting Pol I to the promoter region [29]. In the analysis, only orthologs were found for the POLR1F subunit, thus all intestinal protozoan parasites lacking identifiable RPA3 orthologs. RPA3 prevents DNA rehybridization during transcription and, in parallel, recruits and activates different proteins and complexes [30]. For proteins in which orthologs were not found, it does not necessarily mean that these are not present in these organisms. It may be that their identity is very low and a special search is required to find them, as was the case with RRN3 of *E. histolytica* [31]. Using the BLAST tool with default search parameters, RRN3 orthologs were found in the genome of *G. duodenalis* and *C. parvum*, but not in the genomes of *E. histolytica* and *E. dispar*. However, previously Srivastava *et al*. [31] reported a putative *E. histolytica* ortholog of RRN3 and of which there is a homologue in *E. dispar*. Differences in the components of the rRNA transcription machinery may be due to differences in the promoter, how the rDNA is organized in the genome of the parasites, and the number of copies, among other things. The way in which rDNA is organized in intestinal protozoan parasites varies between species, for *G. duodenalis* and *C. parvum* the classical conformation of repeats in tandem is maintained [32, 33], but for *E. histolytica*, these genes are located on extrachromosomal circular DNA molecules [34]. Regarding the number of rRNA copies, *G. duodenalis* has approximately 86 copies [32], *C. parvum* 5 copies [33] and *E. histolytica* approximately 200 copies [35]. Furthermore, the rDNA promoters of *G. duodenalis* and *C. parvum* have not been identified, but that of *E. histolytica* has [36]. Interestingly, in *G. duodenalis*, the presence of binding sequences for TBP and TAF in the intergenic region of the rDNA were identified [8].

The interaction between RRN3 and the A43 subunit (POLR1F in humans) is essential for the recruitment of Pol I into the preinitiation complex in the rDNA promoter [37, 38]. Important to this interaction is a conserved region of 22 amino acids in A43 [37] and a conserved serine patch on the surface of RRN3 which is formed by residues S101, S102, S109, S110, S145, S146, S185 and S186 [39]. This interaction is also regulated by phosphorylation of both proteins [39, 40]. In the conducted research, both regions were found to be partially conserved in intestinal protozoan parasites, with 8 residues (out of 22) highly conserved in the A43 counterparts and the residues corresponding to S101, S102 and S185 in the RRN3 counterparts (Figs. 2A, 3A). There are currently two drugs in cancer clinical trials that target the RNA polymerase I transcription (CX-5461 and CX-3543) and, in particular, CX-5461 does this by preventing the interaction between SL1 and Pol I in the rRNA promoter [41]. In this way, based on our analysis, molecules similar to CX-5461 could be designed against intestinal parasite rRNA transcription machineries as a new treatment strategy. Although it should also be considered that the subspecies and variants of the mentioned protozoa may have differences in sequence and structure. Given the differences between human and parasitic proteins, it may be possible to design molecules that specifically inhibit this machinery in parasites (and thus not affect the hosts), where the residues and regions that stand out in this work can be taken as a starting point.

## Supplementary Information

Below is the link to the electronic supplementary material.

Supplementary file1 Size and domains organization of conserved subunits in intestinal parasites of the rRNA transcription machinery. TBP (A), RRN3 (B), POLR1A (C), POLR1B (D), POLR1C (E), POLR1D (F), POLR1F (G), POLR1H (H), POLR2E (I), POLR2F (J), and POLR2H (K) (TIF 2638 KB)

## References

[1] Theel ES, Pritt BS (2016). Parasites. Microbiol Spectr.

[2] El-Taweel HA (2015). Understanding drug resistance in human intestinal protozoa. Parasitol Res.

[3] Hemphill A, Müller N, Müller J (2019). Comparative pathobiology of the intestinal protozoan parasites *Giardia lamblia*, *Entamoeba histolytica*, and *Cryptosporidium parvum*. Pathogens.

[4] Chifunda K, Kelly P (2019). Parasitic infections of the gut in children. Paediatr Int Child Health.

[5] Pramanik PK, Alam MN, Roy Chowdhury D, Chakraborti T (2019). Drug resistance in protozoan parasites: an incessant wrestle for survival. J Glob Antimicrob Resist.

[6] Upcroft P, Upcroft JA (2001). Drug targets and mechanisms of resistance in the anaerobic protozoa. Clin Microbiol Rev.

[7] Grummt I (2013). The nucleolus—guardian of cellular homeostasis and genome integrity. Chromosoma.

[8] Lagunas-Rangel FA, Bazán-Tejeda ML, Bermúdez-Cruz RM (2021). Ribosomal DNA in the protozoan parasite *Giardia duodenalis* has a differential chromatin distribution and epigenetic markings across the subunits. Acta Trop.

[9] Lagunas-Rangel FA, Yee J, Bermúdez-Cruz RM (2021). An update on cell division of *Giardia duodenalis* trophozoites. Microbiol Res.

[10] Mancio-Silva L, Lopez-Rubio JJ, Claes A, Scherf A (2013). Sir2a regulates rDNA transcription and multiplication rate in the human malaria parasite *Plasmodium falciparum*. Nat Commun.

[11] McStay B, Grummt I (2008). The epigenetics of rRNA genes: from molecular to chromosome biology. Annu Rev Cell Dev Biol.

[12] Lagunas-Rangel FA, Yee J, Bazán-Tejeda ML (2021). Sirtuin GdSir2.4 participates in the regulation of rRNA transcription in the *Giardia duodenalis* parasite. Mol Microbiol.

[13] Russell J, Zomerdijk JCBM (2006). The RNA polymerase I transcription machinery. Biochem Soc Symp.

[14] Bakari-Soale M, Ikenga NJ, Scheibe M (2021). The nucleolar DExD/H protein Hel66 is involved in ribosome biogenesis in *Trypanosoma brucei*. Sci Rep.

[15] Jaremko D, Ciganda M, Christen L, Williams N (2019). *Trypanosoma brucei* L11 is essential to ribosome biogenesis and interacts with the kinetoplastid-specific proteins P34 and P37. mSphere.

[16] Nepomuceno-Mejía T, Florencio-Martínez LE, Pineda-García I, Martínez-Calvillo S (2022). Identification of factors involved in ribosome assembly in the protozoan parasite Leishmania major. Acta Trop.

[17] Lagunas-Rangel FA, Bazán-Tejeda ML, García-Villa E, Bermúdez-Cruz RM (2020). Nicotinamide induces G2 cell cycle arrest in *Giardia duodenalis* trophozoites and promotes changes in sirtuins transcriptional expression. Exp Parasitol.

[18] Bateman A, Martin M-J, Orchard S (2021). UniProt: the universal protein knowledgebase in 2021. Nucleic Acids Res.

[19] Aurrecoechea C, Barreto A, Basenko EY (2017). EuPathDB: the eukaryotic pathogen genomics database resource. Nucleic Acids Res.

[20] Camacho C, Coulouris G, Avagyan V (2009). BLAST+: architecture and applications. BMC Bioinform.

[21] Blum M, Chang H-Y, Chuguransky S (2021). The InterPro protein families and domains database: 20 years on. Nucleic Acids Res.

[22] Waterhouse A, Bertoni M, Bienert S (2018). SWISS-MODEL: homology modelling of protein structures and complexes. Nucleic Acids Res.

[23] Pettersen EF, Goddard TD, Huang CC (2004). UCSF Chimera––a visualization system for exploratory research and analysis. J Comput Chem.

[24] Srivastava R, Srivastava R, Ahn SH (2016). The epigenetic pathways to ribosomal DNA silencing. Microbiol Mol Biol Rev.

[25] Sharifi S, Bierhoff H (2018). Regulation of RNA polymerase I transcription in development, disease, and aging. Annu Rev Biochem.

[26] Kramm K, Engel C, Grohmann D (2019). Transcription initiation factor TBP: old friend new questions. Biochem Soc Trans.

[27] Sanij E, Hannan RD (2009). The role of UBF in regulating the structure and dynamics of transcriptionally active rDNA chromatin. Epigenetics.

[28] Kuhn C-D, Geiger SR, Baumli S (2007). Functional architecture of RNA polymerase I. Cell.

[29] Peyroche G, Levillain E, Siaut M (2002). The A14–A43 heterodimer subunit in yeast RNA pol I and their relationship to Rpb4-Rpb7 pol II subunits. Proc Natl Acad Sci.

[30] Imazawa Y, Hisatake K, Mitsuzawa H (2005). The fission yeast protein Ker1p is an ortholog of RNA polymerase I subunit A14 in *Saccharomyces cerevisiae* and is required for stable association of Rrn3p and RPA21 in RNA polymerase I. J Biol Chem.

[31] Srivastava A, Bhattacharya A, Bhattacharya S, Jhingan GD (2016). Identification of EhTIF-IA: the putative *E. histolytica* orthologue of the human ribosomal RNA transcription initiation factor-IA. J Biosci.

[32] Xu F, Jex A, Svärd SG (2020). A chromosome-scale reference genome for *Giardia intestinalis* WB. Sci Data.

[33] Le Blancq SM, Khramtsov NV, Zamani F (1997). Ribosomal RNA gene organization in *Cryptosporidium parvum*. Mol Biochem Parasitol.

[34] Bhattacharya S, Som I, Bhattacharya A (1998). The ribosomal DNA plasmids of entamoeba. Parasitol Today.

[35] Torres-Machorro AL, Hernández R, Cevallos AM, López-Villaseñor I (2010). Ribosomal RNA genes in eukaryotic microorganisms: witnesses of phylogeny?. FEMS Microbiol Rev.

[36] Panigrahi SK, Jhingan GD, Som I (2009). Promoter analysis of palindromic transcription units in the ribosomal DNA circle of *Entamoeba histolytica*. Eukaryot Cell.

[37] Rothblum K, Hu Q, Penrod Y, Rothblum LI (2014). Selective inhibition of rDNA transcription by a small-molecule peptide that targets the interface between RNA polymerase I and Rrn3. Mol Cancer Res.

[38] Peyroche G (2000). The recruitment of RNA polymerase I on rDNA is mediated by the interaction of the A43 subunit with Rrn3. EMBO J.

[39] Blattner C, Jennebach S, Herzog F (2011). Molecular basis of Rrn3-regulated RNA polymerase I initiation and cell growth. Genes Dev.

[40] Cavanaugh AH, Hirschler-Laszkiewicz I, Hu Q (2002). Rrn3 phosphorylation is a regulatory checkpoint for ribosome biogenesis. J Biol Chem.

[41] Ferreira R, Schneekloth JS, Panov KI (2020). Targeting the RNA polymerase I transcription for cancer therapy comes of age. Cells.

