# Supplementary figures and images for: Ribosomal RNA Transcription Machineries in Intestinal Protozoan Parasites: A Bioinformatic Analysis
Source: Acta Parasitol. 2022 Aug 27;67(4):1788–99. doi: 10.1007/s11686-022-00612-7 (PMC9705513; doi:10.1007/s11686-022-00612-7)

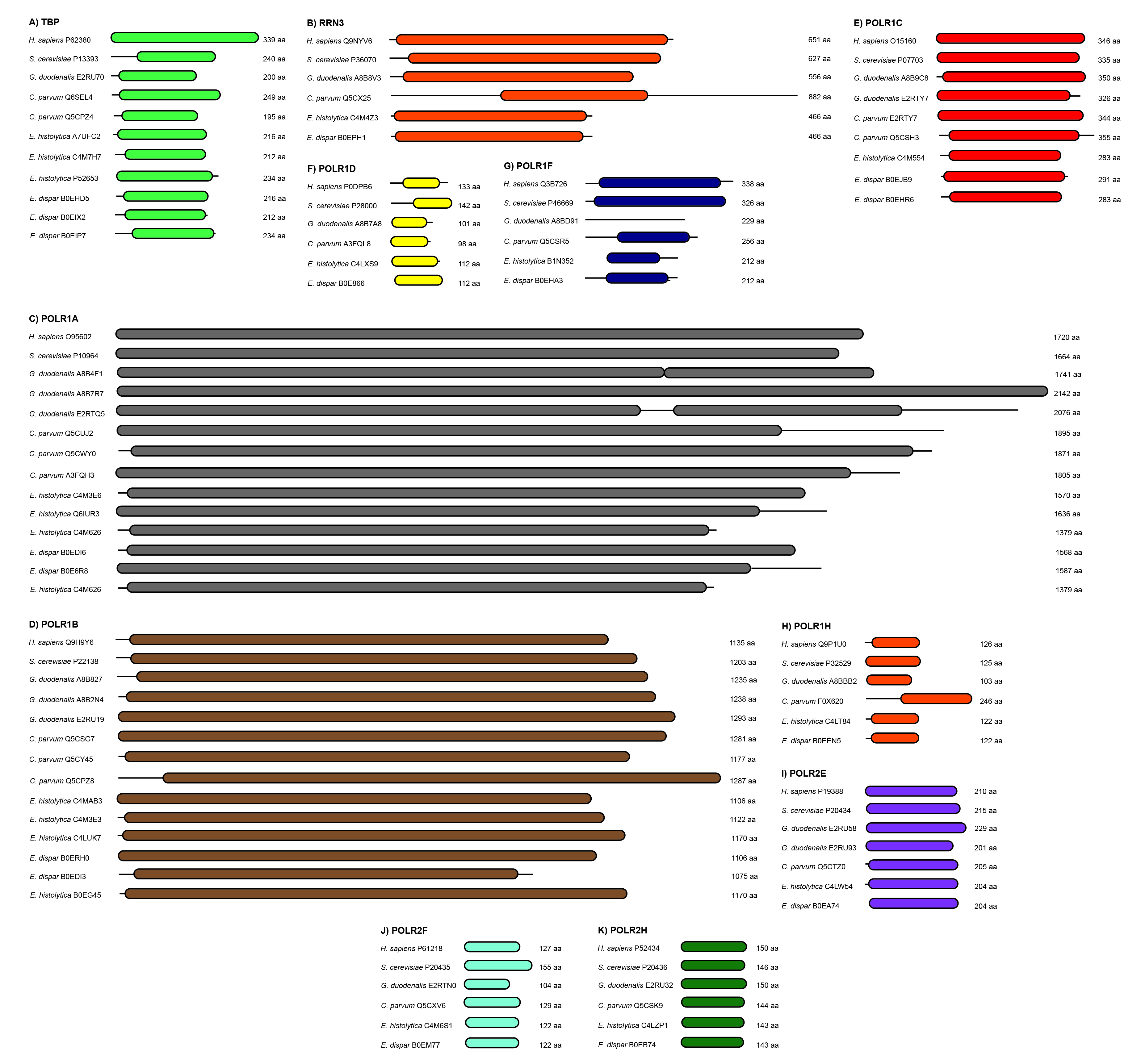

Supplement: Supplementary file 1 — Supplementary file1 Size and domains organization of conserved subunits in intestinal parasites of the rRNA transcription machinery. TBP (A), RRN3 (B), POLR1A (C), POLR1B (D), POLR1C (E), POLR1D (F), POLR1F (G), POLR1H (H), POLR2E (I), POLR2F (J), and POLR2H (K) (TIF 2638 KB) [file 11686_2022_612_MOESM1_ESM.tif]
